# Diagnostic Methods for Detecting Dental Cracks: A Systematic Review

**DOI:** 10.3390/dj14080482

**Published:** 2026-08-05

**Authors:** Beatriz Hernando Dumaraog, Yasser Shehata Elsayed, Vicente Vera González, Vicente Vera-Rodríguez, Diego González-Gil

**Affiliations:** 1 Department of Conservative Dentistry and Prosthesis, Faculty of Dentistry, Complutense University, 28040 Madrid, Spain; beaher04@ucm.es (B.H.D.); vveragon@ucm.es (V.V.G.); 2 Faculty of Dentistry, Complutense University, 28040 Madrid, Spain; 3 Center for Implant Dentistry, University of Florida, Gainesville, FL 32610, USA; vicevera@ucm.es; 4 Department of Surgery, Faculty of Medicine, Dental Clinic, University of Salamanca, 37007 Salamancam, Spain; diegoggil@usal.es

**Keywords:** dental crack, cracked tooth syndrome, diagnosis, diagnostic accuracy, cracked tooth, imaging, detection

## Abstract

**Background:** Dental cracks are a common yet diagnostically challenging condition due to their asymptomatic clinical nature and difficulty in early detection. Accurate diagnosis is essential to prevent crack progression and potential tooth loss; however, no universally accepted diagnostic method exists. **Objectives:** To systematically evaluate the diagnostic methods used for detecting dental cracks and analyze their effectiveness and limitations according to the available scientific literature. **Materials and Methods:** A systematic review of diagnostic test accuracy studies was conducted following the PRISMA 2020 guidelines in the PubMed and SCOPUS academic databases using the following keywords and Boolean operators *(“cracked tooth” OR “cracked tooth syndrome” OR “tooth cracks” OR “dental cracks” OR “enamel cracks”) AND (diagnosis OR diagnostic OR detection OR imaging OR “diagnostic accuracy”).* The search included studies published within the last 10 years, written in English, and with free full-text availability. Diagnostic test accuracy studies evaluating clinical or imaging methods for detecting dental cracks were included. Study selection, data extraction, and risk-of-bias assessment using the QUADAS-2 tool were performed independently by two reviewers. Owing to methodological heterogeneity, a qualitative synthesis of the evidence was undertaken. **Results:** The literature search identified 81 records, of which 12 studies met the eligibility criteria and were included in the systematic review. The evaluated diagnostic methods included visual examination, transillumination, quantitative light-induced fluorescence, cone-beam computed tomography, contrast-enhanced cone-beam computed tomography, optical coherence tomography, magnetic resonance imaging, and artificial intelligence-assisted image analysis. Considerable variability in diagnostic performance was observed among the evaluated methods. Optical coherence tomography demonstrated the highest diagnostic accuracy for detecting enamel and dentinal cracks, whereas contrast-enhanced cone-beam computed tomography improved crack detection compared with conventional cone-beam computed tomography. Conventional clinical methods remained useful as initial screening tools but showed limited diagnostic accuracy when used alone. **Conclusions:** No single diagnostic method demonstrated consistently high diagnostic accuracy for all types of dental cracks. Current evidence supports a multimodal diagnostic approach that combines conventional clinical examination with advanced imaging techniques when indicated to improve diagnostic reliability and facilitate treatment planning. However, the available evidence remains heterogeneous, highlighting the need for additional high-quality diagnostic test accuracy studies using standardized reference standards and reporting methods.

## 1. Introduction

In dentistry, a dental crack is defined as an incomplete fracture within the tooth structure that may extend through the enamel and dentin without complete separation of the tooth. This crack generally extends from the crown toward the gingiva and may involve the pulp or the surrounding periodontal tissues [1].

The prevalence of dental cracks in adults has been reported to reach up to 70%, although differences exist in the morphology and location of cracks among individuals. Dentists often encounter difficulties diagnosing cracks in the early stages due to the absence of clear signs and symptoms [2].

Most studies report that cracks occur primarily in restored teeth, while non-restored teeth account for only 35% of cracked teeth [3].

The etiology of dental cracks is multifactorial. Masticatory forces are considered the most common factor associated with vertical tooth fractures. As a result, the highest prevalence of cracked teeth is reported in patients around the age of 50 [4].

The diagnosis of dental cracks remains one of the greatest challenges in restorative dentistry and endodontics. Cracks are frequently asymptomatic, which prevents patients from seeking dental treatment [1,5]. Additionally, the selection of an appropriate treatment may be complicated due to diagnostic difficulties and the variability of symptoms depending on the direction and rate of crack progression [6,7].

The clinical presentation of dental cracks is highly variable, further complicating their diagnosis and management. Although many cracks remain asymptomatic, others may cause intermittent pain during chewing or sensitivity to thermal stimuli [8].

According to the American Association of Endodontists (AAE), longitudinal tooth fractures are classified into five types: craze lines, fractured cusp, cracked tooth, split tooth, and vertical root fracture.

It is important to note that this classification does not address the internal extension or termination of cracks, which are important factors when determining prognosis and selecting a treatment plan for cracked teeth [9].

Alternative classifications, such as that proposed by Imai et al. [10], incorporate crack depth and allow better prediction of structural compromise. which consists of 5 types:

1: an intact surface with no crack;
2: a superficial enamel crack (an enamel crack < 50% of enamel thickness);
3: a deep enamel crack (an enamel crack > 50% of enamel thickness but without extending to the amelodentinal junction);
4: a full-thickness enamel crack (an enamel crack of 100% that extends to the DEJ); and
5: a dentinal crack (a crack that extends beyond the DEJ).

Several diagnostic methods have been used to detect dental cracks, including visual examination, methylene blue dye application, microscopic examination, fiber-optic transillumination (FOTI), bite test, radiographic examinations including cone-beam computed tomography (CBCT) and optical coherence tomography imaging (SS-OCT) [10,11]. 

Studies evaluating visual diagnostic techniques have shown that commonly used clinical methods may only achieve moderate diagnostic accuracy and may require combination with other diagnostic approaches to improve reliability [12].

Despite the availability of these diagnostic approaches, no universal method for detecting dental cracks has been established. Additionally, treatment decisions remain inconsistent among clinicians due to differences in diagnostic interpretation [13].

Given the diagnostic challenges associated with dental cracks, the variability in the reported accuracy of available diagnostic methods due to differences in study design, reference standards, and study populations, and the potential consequences of an incorrect diagnosis, a comprehensive synthesis of the available literature is warranted. Therefore, the aim of this systematic review is to evaluate the current scientific evidence on dental cracks, with a particular focus on their risk factors, diagnostic methods and their diagnostic accuracy, and clinical outcomes, in order to support evidence-based clinical decision-making.

## 2. Materials and Methods

### 2.1. Study Design

The search strategy was developed using the following population, intervention, comparison, and outcome (PICO) framework:

Population (P): Teeth with suspected or diagnosed dental cracks or with diagnosed dental cracks.

Intervention (I): Diagnostic methods used for crack detection.

Comparison (C): Comparison between different diagnostic modalities or reference standards.

Outcome (O): Diagnostic accuracy, sensitivity, specificity, and crack detection capability.

Based on the following question: “In patients or extracted human teeth with suspected dental cracks, how do different diagnostic methods compare in terms of accuracy when evaluated against a gold standard?”

### 2.2. Search Strategy

A comprehensive electronic literature search was conducted on the 1st of February in the year 2026 using the PubMed/MEDLINE and Scopus library databases to identify relevant studies published during the previous 10 years. The search strategy combined keywords, MeSH terms, and Boolean operators related to dental cracks and diagnostic methods.

The following Boolean operators were used *(“cracked tooth” OR “cracked tooth syndrome” OR “tooth cracks” OR “dental cracks” OR “enamel cracks”) AND (diagnosis OR diagnostic OR detection OR imaging OR “diagnostic accuracy”).* The search only included studies published during the previous 10 years, written in English, and with free full-text availability by applying the necessary filter in each database.

This systematic review was conducted in accordance with the Preferred Reporting Items for Systematic Reviews and Meta-Analyses (PRISMA 2020) guidelines. The review aimed to evaluate and compare currently available diagnostic methods for the detection of dental cracks, including conventional clinical techniques, radiographic imaging, and advanced diagnostic technologies. This study is registered within the PROSPERO database under the following registration code: CRD420261357857 (Supplementary Material Table S1).

### 2.3. Inclusion Criteria

1. Studies that evaluated diagnostic methods for the detection of dental cracks or cracked tooth syndrome
2. Studies including reported diagnostic performance outcomes such as accuracy, sensitivity, or specificity
3. In vitro, clinical, observational, or experimental study designs

### 2.4. Exclusion Criteria

1. Studies that are review articles, case reports, editorials, conference abstracts, or letters to the editor
2. Studies that did not primarily evaluate diagnostic methods for dental cracks
3. Studies that lacked sufficient diagnostic outcome data

### 2.5. Study Selection

All identified studies were imported into a reference management system, and duplicate records were removed. Titles and abstracts were initially screened by one reviewer and independently verified by a second reviewer. Any discrepancies were resolved through discussion. Articles that were potentially eligible were then selected for full-text review to be analyzed according to their relevance to the objectives of this study.

### 2.6. Data Extraction

Data extraction was performed using a standardized extraction form. The characteristics of the included studies were extracted and summarized in 2 tables: the first table included study type, diagnostic methods, reference standards, results and conclusions, while the other table included accuracy, sensitivity and specificity.

Data extraction was performed by one reviewer using a standardized data extraction form and subsequently verified by a second reviewer to ensure accuracy and consistency. Any discrepancies identified during the verification process were resolved through discussion until consensus was reached.

### 2.7. Risk of Bias Assessment

The methodological quality of the included studies was assessed using the QUADAS-2 (Quality Assessment of Diagnostic Accuracy Studies 2) tool. The studies were evaluated across four domains:

- Sample selection
- Index test
- Reference standard
- Flow and timing
- Overall Risk

### 2.8. Data Synthesis

Due to heterogeneity among the included studies in terms of study design, diagnostic modalities, reference standards, and reported outcomes, a meta-analysis was not performed.

The diagnostic methods were compared according to their reported accuracy, sensitivity, specificity, advantages, and limitations. Particular emphasis was placed on comparing conventional diagnostic methods with advanced imaging technologies such as CBCT, optical coherence tomography, and artificial intelligence-based approaches.

## 3. Results

**Figure 1:**
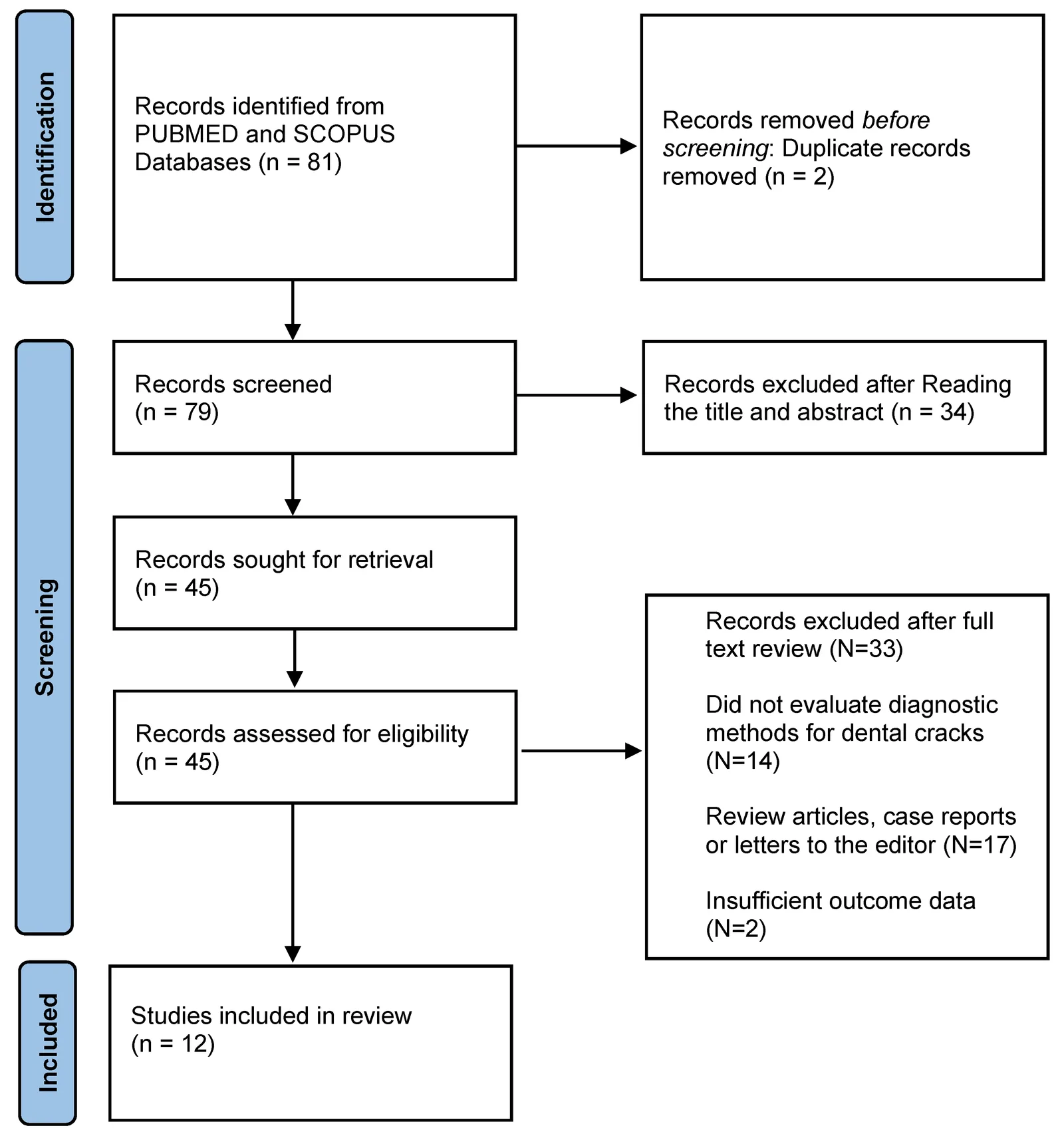
Flowchart showing the selection process for the systematic review.

The electronic search yielded 81 studies (74 from PubMed and 7 from Scopus). 2 Duplicates were removed, and after reviewing the titles and abstracts, the number was reduced to 45 studies for full-text review. Following additional screening, 33 studies were excluded; 17 of these studies were excluded as they were review articles, case reports, or letters to the editor; meanwhile 14 studies were excluded as they did not primarily evaluate diagnostic methods for dental cracks and, finally, 2 studies were excluded as they lacked sufficient outcome data, leaving 12 studies that met the eligibility criteria and were included in this study. This process is illustrated in Figure 1. A total of 12 studies were included in this systematic review. The characteristics of the included studies were systematically extracted and presented in Table 1, including study type, diagnostic methods, reference standards, results, and conclusions. The diagnostic performances of all the modalities that were used in the selected articles were evaluated by including accuracy, sensitivity, and specificity, as shown in Table 2.

The QUADAS-2 assessment presented in Table 3 showed that most included studies had a low to unclear risk of bias. Established diagnostic methods such as CBCT, OCT, microscopy, and transillumination generally demonstrated low risk across most domains, while studies involving newer technologies, particularly artificial intelligence-based systems, showed more unclear risk due to limited reporting of sample selection and validation methods. Overall, the included studies were considered methodologically acceptable, although variations in study design and reporting quality may affect the generalizability of the findings.

Considerable variability was observed among the evaluated diagnostic methods. Light-based techniques such as transillumination, DIAGNOcam, and QLF generally demonstrated moderate diagnostic performance, with reported accuracy values ranging from approximately 64% to 70%. These methods showed relatively high sensitivity but lower specificity, indicating their usefulness as screening tools rather than definitive diagnostic modalities.

Conventional radiographic techniques, including CBCT and periapical radiography, demonstrated inconsistent diagnostic performance across studies. Standard CBCT showed limited sensitivity for detecting fine or early-stage cracks, whereas contrast-enhanced CBCT demonstrated improved crack visualization and diagnostic accuracy. However, the effectiveness of CBCT remained dependent on x-ray direction, crack orientation, depth, and imaging resolution.

Among all evaluated methods, optical coherence tomography (OCT), particularly swept-source OCT, demonstrated the highest overall diagnostic performance, with superior sensitivity and specificity compared with transillumination and conventional imaging methods. MRI-based approaches also showed promising diagnostic potential, although evidence remains limited.

Emerging technologies such as artificial intelligence-based image analysis demonstrated encouraging preliminary results for automated crack detection. However, these approaches remain experimental and require further clinical validation before routine implementation.

## 4. Discussion

The diagnosis of dental cracks remains a complex clinical challenge, as demonstrated by the findings of the included studies. The variability in diagnostic accuracy across different methods reflects the effect of the multifactorial nature of crack detection, which depends on factors such as crack size, location, and depth [25,26]. This systematic review highlights that no single diagnostic method is capable of consistently identifying dental cracks with high accuracy, particularly in their early stages.

Overall, the included studies indicate that conventional clinical methods remain essential for the initial assessment of suspected dental cracks because of their accessibility and ease of use. However, advanced imaging techniques generally demonstrated greater diagnostic capability, particularly for detecting early or subsurface cracks, although their clinical implementation remains limited by cost, availability, and technical requirements. Conventional diagnostic methods, including visual inspection, continue to be widely used in clinical practice due to their simplicity and accessibility. However, their limitations are evident. Hausdörfer et al. [17] reported only moderate diagnostic accuracy using visual methods, with significant inter-examiner variability. Similarly, the findings discussed by Shimada et al. [20] emphasize that early cracks often present with minimal or no visible signs, making visual diagnosis unreliable. These limitations are further compounded in clinical settings, where lighting conditions, operator experience, and patient-related factors may influence detection. Therefore, while visual inspection remains a crucial diagnostic method, it cannot be relied upon as a definitive diagnostic method [27]. Consequently, conventional clinical examination should be considered an initial screening approach and, whenever clinical uncertainty persists, should be complemented by additional diagnostic methods.

Light-based diagnostic techniques have been developed to address some of these limitations. Quantitative light-induced fluorescence (QLF), as evaluated by Son et al. [14], Lee et al. [16], and Jun et al. [24], demonstrated improved detection rates and high inter-examiner reliability compared with conventional visual methods. These findings suggest that QLF enhances the visualization of structural changes in enamel, making it more effective for identifying early-stage cracks, which could be supported by previous literature from Kühnisch et al. [28]. Additionally, Zheng et al. [29] introduced an infrared imaging system that provided enhanced visualization of enamel cracks with a relatively low-cost approach. Despite these advantages, both QLF and infrared imaging remain limited in their ability to assess crack depth and subsurface propagation, which are critical factors in determining prognosis and treatment planning. Therefore, although QLF and infrared imaging improve the detection of superficial enamel cracks, they should be regarded as complementary diagnostic tools rather than standalone methods.

Radiographic imaging, particularly CBCT, offers three-dimensional visualization and has been investigated extensively in the included studies. However, the results indicate inconsistent diagnostic performance. Both Hu et al. [19] and Wang et al. [23] reported that conventional CBCT and periapical radiography have limited sensitivity in detecting fine or early cracks. This limitation is primarily due to the resolution constraints of CBCT and the dependence on crack orientation relative to the imaging plane [30]. To overcome these challenges, contrast-enhanced CBCT has been proposed. Studies by Hu et al. [15] and Zhou et al. [18] demonstrated that contrast enhancement improves crack detection and allows for better assessment of crack depth. Nevertheless, these improvements are still influenced by crack characteristics and may not be sufficient for detecting microcracks. Furthermore, the use of contrast agents may limit routine clinical applicability.

Advanced imaging modalities, particularly optical coherence tomography (OCT), demonstrated the highest diagnostic potential among the included studies. Kim et al. [22] reported high sensitivity using automated OCT detection, highlighting its ability to identify cracks that are not visible with conventional methods. Similarly, Shimada et al. [20] emphasized the capability of OCT to provide high-resolution cross-sectional images, allowing detailed visualization of crack propagation within dental tissues. This indicates that OCT addresses many of the limitations associated with both visual and radiographic methods. Although OCT demonstrated superior diagnostic performance, its limited accessibility currently restricts its widespread adoption in routine dental practice, emphasizing the need for further technological development and cost reduction before broader clinical implementation.

Other advanced imaging modalities demonstrated variable diagnostic performance across the included studies. Schuurmans et al. [21] reported that magnetic resonance imaging (MRI) showed promising capability for detecting root cracks and fractures without ionizing radiation exposure. Although MRI demonstrated potential as a noninvasive diagnostic alternative, limitations related to cost, accessibility, and image resolution currently restrict its routine clinical application in dentistry. Therefore, MRI currently represents a promising adjunctive diagnostic modality rather than a replacement for conventional clinical or radiographic examination.

The importance of a multimodal diagnostic approach is strongly supported by the findings of Kindaro et al. [12], who compared multiple diagnostic techniques and demonstrated that methods such as transillumination provide high sensitivity, while others such as macrophotography and microscopy offer higher specificity. This highlights the complementary nature of different diagnostic tools. Combining methods allows clinicians to balance sensitivity and specificity, thereby improving overall diagnostic accuracy. This concept is consistent with established clinical recommendations that emphasize the use of multiple diagnostic tools to improve diagnostic accuracy in cracked tooth cases [31].

A key observation across the included studies is the frequent use of micro-computed tomography (micro-CT) as the reference standard in in vitro research. Micro-CT provides high-resolution imaging capable of detecting microstructural defects with great precision [32]. However, its application is limited to laboratory settings and cannot be used in routine clinical practice. This creates a significant gap between experimental findings and real-world applicability, emphasizing the need for clinically feasible diagnostic tools that approach the accuracy of micro-CT.

Several limitations of this systematic review should be acknowledged. The search was restricted to the PubMed and Scopus databases and to free full-text availability during the past 10 years to have access to the data so it can be included in the study and to include diagnostic methods that are up to date, which may have resulted in the omission of relevant studies. The majority of the included studies were conducted in vitro, which may not accurately replicate clinical conditions. Additionally, heterogeneity in study design, diagnostic methods, and outcome measures limited direct comparison between studies and prevented the performance of a meta-analysis. Variations in sample size, operator experience, and evaluation criteria may have further influenced the reported outcomes.

Future research should focus on well-designed clinical studies that evaluate diagnostic methods under real clinical conditions. Standardization of diagnostic criteria and outcome measures is essential to improve comparability across studies. Furthermore, continued development of advanced imaging technologies and AI-based tools may play a critical role in improving the early detection and management of dental cracks.

## 5. Clinical Implications

From a clinical perspective, the findings of this review suggest that no single diagnostic method is sufficient for reliable crack detection under all clinical conditions. Conventional methods such as visual inspection and transillumination remain useful as a method for primary diagnosis due to their simplicity, accessibility, and low cost. However, their limited specificity may increase the risk of false-positive diagnoses, particularly in cases involving superficial enamel defects.

Advanced imaging modalities such as OCT demonstrated substantially improved diagnostic performance and may be particularly valuable in complex or uncertain cases where conventional methods fail to provide definitive findings. Nevertheless, routine clinical implementation of OCT remains limited due to high equipment cost, limited availability, and the need for specialized training.

Although contrast-enhanced CBCT improved crack visualization compared with conventional CBCT and conventional periapical X-rays, it showed limited sensitivity for microcracks that may restrict its routine use for early crack detection. Overall, concerns regarding the use of ionizing methods should be taken into consideration and should be carefully justified due to the radiation exposure that may pose potential biological risks to both patients and dental professionals, particularly with repeated or unnecessary imaging procedures [33]. Therefore, CBCT and other radiographic imaging methods may be more appropriate for evaluating crack extension and associated structural complications rather than initial diagnosis.

Emerging diagnostic technologies continue to demonstrate potential for improving crack detection; however, further clinical validation and standardization are required before routine implementation. However, current evidence remains limited, and further large-scale clinical studies are required before AI-based approaches can be integrated into routine dental practice.

Overall, the findings support a multimodal diagnostic approach that combines conventional clinical examination with advanced imaging techniques when necessary, to improve diagnostic accuracy and treatment planning.

## 6. Conclusions

- The diagnosis of dental cracks remains challenging due to the variability in diagnostic accuracy among the available methods.
- No single diagnostic method is sufficient; therefore, a multimodal approach is recommended to improve diagnostic reliability.
- Advanced imaging modalities, especially optical coherence tomography (OCT), demonstrate the highest diagnostic accuracy but are limited by their cost and clinical availability.
- Further well-designed clinical studies and standardized diagnostic protocols are needed to improve the diagnosis and management of dental cracks.

## Figures and Tables

**Table 1:** Showing the comparison of the articles that have been selected.

Study	Study Type	Diagnostic Methods Used	Reference Standard	Main Findings	Clinical Interpretation
Kindaro et al. (2025) [12]	In vitro	Macrophotography Transillumination DIAGNOcam Surgical Microscope	Microcomputed tomography (Micro-CT)	Transillumination showed the highest sensitivity and overall accuracy, whereas macrophotography and microscopy demonstrated the highest specificity.	A multimodal diagnostic approach improves crack detection.
Son et al. (2024) [14]	In vivo	Quantitative light-induced fluorescent imaging (QLF)	Clinical Examination	QLF improved crack detection and showed high inter-examiner agreement.	QLF is a useful adjunct but should not replace clinical examination.
Hu et al. (2024) [15]	In vitro	Contrast-enhanced CBCT	Clinically diagnosed extracted teeth	Contrast-enhanced CBCT detected more cracks than conventional CBCT but remained limited for very fine cracks.	Contrast enhancement improves CBCT performance.
Lee et al. (2023) [16]	In vivo	Quantitative light-induced fluorescence (QLF)	Clinical diagnosis	No significant difference was observed between symptomatic and contralateral teeth using QLF parameters.	QLF alone is insufficient for diagnosis.
Hausdörfer et al. (2023) [17]	In vitro	Surgical Microscope FOTI DIAGNOcam	Histological horizontal sectioning	FOTI showed the highest diagnostic performance among visual methods, although crack depth could not be estimated accurately.	Suitable for crack detection but not depth assessment.
Zhou et al. (2022) [18]	In vitro	CBCT Contrast-enhanced CBCT	Artificially simulated cracked teeth with Micro-CT to measure crack depth	Contrast-enhanced CBCT detected more cracks than conventional CBCT but did not accurately determine crack depth.	Improves detection but not depth evaluation.
Hu et al. (2021) [19]	In vitro	CBCT (RS) CBCT with meglumine diatrizoate as contrast agent. (ES1) CBCT with NaI + DMSO as contrast agent (ES2) Micro-CT	Artificially simulated cracked teeth	Contrast-enhanced CBCT with NaI/DMSO showed diagnostic performance comparable to Micro-CT.	Selected contrast agents substantially improve CBCT accuracy.
Shimada et al. (2020) [20]	In vitro	Transillumination Swept source optical coherence tomography (SS-OCT)	Observer-based scoring (visual assessment by three dentists)	SS-OCT demonstrated higher sensitivity and reproducibility than transillumination.	SS-OCT offers superior diagnostic performance.
Schuurmans et al. (2019) [21]	In vitro	Magnetic resonance imaging (MRI)	CBCT imaging	MRI showed diagnostic performance comparable to CBCT but requires further technological development.	MRI has potential but is not yet suitable for routine use.
Kim et al. (2017) [22]	In vitro	SS-OCT Intraoral X-ray Transillumination CBCT	Artificially simulated cracked teeth	SS-OCT detected early cracks that conventional methods failed to identify.	SS-OCT is superior for early crack detection.
Wang et al. (2017) [23]	In vitro	CBCT Periapical radiography	Artificially simulated cracked teeth with Micro-CT to measure crack depth	Both radiographic techniques showed limited sensitivity for fine cracks.	Conventional radiography is inadequate for early diagnosis.
Jun et al. (2016) [24]	In vitro	Quantitative light-induced fluorescence (QLF)	Histological sectioning	QLF demonstrated excellent performance for detecting early enamel cracks.	QLF is valuable for early enamel crack detection.

**Table 2:** Showing the comparison between the diagnostic performance between the modalities used in all the articles. NR; Not Reported.

Study	Sample Size	Diagnostic Methods	Accuracy %	Sensitivity %	Specificity %
Kindaro et al. (2025) [12]	N = 30	Diagnocam	64.4	65.4	60.7
		Transillumination	65.3	68.8	53.6
		High magnification microscope	62.5	54.6	92.9
		Macrophotography	60.6	51.9	92.9
Son et al. (2024) [14]	N = 81	QLF	NR	NR	NR
Hu et al. (2024) [15]	N = 14	CBCT with different contrast agents	NR	NR	NR
Lee et al. (2023) [16]	N = 90	QLF	NR	96.0	NR
Hausdörfer et al. (2023) [17]	N = 89	High Magnification Microscope	56.9	29.25	81.00
		Transilumination	69.6	37.25	89.00
		Diagnocam	67.2	30.75	91.0
Zhou et al. (2022) [18]	N = 31	Pre CBCT	48.4	NR	NR
		Contrast enhanced CBCT	67.7	NR	NR
Hu et al. (2021) [19]	N = 18	CBCT	6.0	NR	NR
		Contrast enhanced CBCT (meglumine diatrizoate)	6.0	NR	NR
		Contrast enhanced CBCT (NaI + DMSO)	71.0	NR	NR
		Micro CT	59.0	NR	NR
Shimada et al. (2020) [20]	N = 20	Transillumination enamel crack	69.0	87.0	50.0
		SS-OCT enamel crack	85.0	95.0	75.0
Schuurmans et al. (2019) [21]	N = 58	MRI	NR	59.0	83.0
		CBCT	NR	59.0	90.0
Kim et al. (2017) [22]	N = 6	SS-OCT	NR	NR	NR
		Transillumination	NR	NR	NR
		CBCT images	NR	NR	NR
		Intraoral radiography images	NR	NR	NR
Wang et al. (2017) [23]	N = 44	CBCT	NR	82.5	NR
Jun et al. (2016) [24]	N = 96	QLF detecting enamel inner half cracks	NR	87.0	98.0
		QLF detecting cracks extending to the DEJ	NR	90.0	100.0

**Table 3:** Risk of bias assessment of included studies using the QUADAS-2 (Quality Assessment of Diagnostic Accuracy Studies-2) tool. 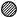 indicates a low risk of bias; 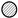 indicates an unclear risk of bias.

Scheme	Section	Index Test	Reference Standard	Flow and Timing	Overall Risk
Kindaro et al. (2025) [12]	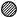 Low	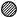 Low	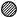 Low	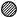 Low	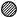 Low
Son et al. (2024) [14]	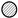 Unclear	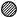 Low	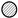 Unclear	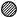 Low	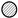 Unclear
Hu et al. (2024) [15]	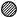 Low	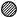 Low	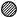 Low	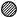 Low	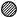 Low
Lee et al. (2023) [16]	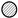 Unclear	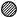 Low	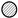 Unclear	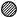 Low	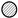 Unclear
Hausdörfer et al. (2023) [17]	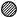 Low	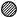 Low	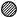 Low	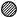 Low	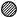 Low
Zhou et al. (2022) [18]	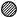 Low	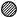 Low	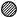 Low	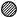 Low	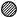 Low
Hu et al. (2021) [19]	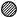 Low	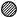 Low	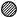 Low	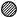 Low	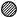 Low
Shimada et al. (2020) [20]	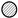 Unclear	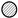 Unclear	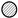 Unclear	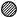 Low	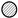 Unclear
Schuurmans et al. (2019) [21]	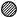 Low	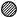 Low	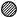 Low	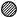 Low	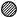 Low
Kim et al. (2017) [22]	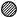 Low	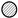 Unclear	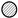 Unclear	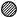 Low	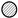 Unclear
Wang et al. (2017) [23]	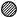 Low	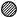 Low	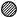 Low	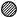 Low	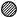 Low
Jun et al. (2016) [24]	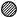 Low	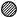 Low	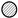 Unclear	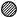 Low	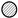 Unclear

## Data Availability

The raw data supporting the conclusions of this article will be made available by the authors on request.

## Supplementary Materials

The following supporting information can be downloaded at: https://www.mdpi.com/article/10.3390/dj14080482/s1, Table S1: The completed PRISMA 2020 checklist.
